# A Peculiar Case

**Published:** 1889-06

**Authors:** H. E. Potter

**Affiliations:** Clifton, Kansas


Clifton, Kansas.

---

### A PECULIAR CASE.

Mrs. G., aet. forty-five years, supposed herself undergoing the "change of life." When I first saw her, November 18th last, she had been flowing about a week—the first for four months. Before I was called she had passed two bodies, which they had not saved, resembling in shape and size the heart of a chicken. To myself I said "mole," and proceeded to arrest the hemorrhage, which was beginning to tell on the patient, who complained of coldness, faintness, and roaring in the ears. Internal medication was first tried but proved ineffectual, either from faulty selection of the remedy or because time enough was not allowed for its action. *Hot* water was next in order, and this was injected directly against the neck of the uterus till a half gallon had been used and the return stream scarcely tinged with blood.

I now left her quite comfortable, the flow having entirely ceased. Next day she was reported "comfortable," but on the following morning she sent in to tell me that she was again flowing slightly, and was in much pain, not having slept at all during the night because of backache. I sent Cimicifuga and visited her about twelve hours later. At this visit a more careful examination was made, revealing the womb in about such condition regarding size and position as would be expected at the close of the fourth month of gestation. I now made a digital examination, finding the os patulous and some stinking debris in the vagina, which latter very much resembled a miniature placenta.

I now determined to empty the uterus of whatever it might contain, so introducing a Brewer speculum I began a careful exploration with the sound. The point of the sound impinged on a yielding body, which was very easily ruptured, a quart or more of fluid escaping having the odor and appearance of amniotic fluid. Well, I expected something to follow this escape of water, and it has a sequel. All pain immediately subsided, and the lady rapidly recovered her usual health and strength under the influence of an occasional dose of Ars. alb. She has menstruated regularly every twenty-eight days since. To me it's something new, and according to the books, I think a very lucky finale.

H. E. Potter, M. D.

